# Immunotherapy in Multiple Myeloma

**DOI:** 10.3390/cells9030601

**Published:** 2020-03-03

**Authors:** Cinnie Yentia Soekojo, Melissa Ooi, Sanjay de Mel, Wee Joo Chng

**Affiliations:** Department of Hematology-Oncology, National University Cancer Institute, Singapore, National University Health System, 1E Kent Ridge Road, Singapore 119228, Singapore; cinnie_yentia_soekojo@nuhs.edu.sg (C.Y.S.); melissa_ooi@nuhs.edu.sg (M.O.); sanjay_widanalage@nuhs.edu.sg (S.d.M.)

**Keywords:** multiple myeloma, immunotherapy, IMiD, monoclonal antibody, ADC, BiTE, checkpoint inhibitor, CAR-T, virotherapy, vaccine

## Abstract

Multiple myeloma is a complex disease and immune dysfunction has been known to play an important role in the disease pathogenesis, progression, and drug resistance. Recent efforts in drug development have been focused on immunotherapies to modify the MM disease process. Here, we summarize the emerging immunotherapies in the MM treatment landscape.

## 1. Introduction

Multiple myeloma (MM) pathogenesis is complex and involves the interaction of MM malignant cells with their microenvironment. Of the many microenvironmental factors, immune surveillance and dysfunction play an important role, and MM cells have been known to evade immune surveillance. Mechanisms implicated in MM immune dysfunction include counterregulatory cells dysfunction, reduced expression of tumor antigenic peptides by the antigen presenting cells, and enhanced inhibitory pathways [1].

Tumour cells can survive in the host when immune surveillance against tumor antigens is impaired [2]. Several mechanisms may contribute to myeloma cell ‘tolerance’, including myeloma-derived cytokines such as transforming growth factor (TGF)-β (which suppresses B cells and T cells via inhibition of IL-2 autocrine pathway [3]), inadequate antigen presentation [4], resistance to NK cell lysis, and defective T, B, natural killer (NK) and NK-T cells [5,6]. Impairment of T-cell activation by dendritic cells (DCs) is also mediated by MM-induced production of TGF-β [5], IL-10 [5], and IL-6 [7], ultimately leading to poor antigen presentation and suboptimal tumor-specific immune response [8]. Inhibitory pathways including the programmed cell death-1 (PD-1)/programmed death ligand-1 (PD-L1) interaction, cytotoxic T-lymphocyte-associated antigen 4 (CTLA-4) and killer cell immunoglobulin-like receptors (KIR) have also been shown to play important roles in MM immune dysfunction.

Hence, the development of immunotherapies with the aim to tackle MM-associated immune dysfunction has generated great interest in recent years. In this review, we will discuss the development of immunotherapies that have emerged in the MM treatment landscape, their promise, challenges and future directions associated with immunotherapy in MM.

## 2. Immunomodulatory Drugs

Immunomodulatory drugs (IMiDs) are a group of compounds that are analogues of thalidomide, with anti-angiogenic properties and potent anti-inflammatory effects due to its anti-tumor necrosis factor (TNF)-α activity. Lenalidomide and pomalidomide, both analogues of thalidomide, entered clinical trials for MM in 1999, and are now the subject of clinical evaluation in other hematological malignancies.

### 2.1. Rationale and Mechanism

T-cell activation requires an antigen-specific T-cell receptor (TCR) signal in conjunction with co-stimulation provided by professional antigen-presenting cells (APCs). IMiDs abrogate the requirement of a secondary co-stimulation signal from APCs to allow T cell activation after the T cells have been partially activated by either anti-CD3 or DC [9]. In the presence of thalidomide, partially activated CD3+ T cells selected from human peripheral blood mononuclear cells (PBMCs) exhibited pronounced proliferation and enhanced production of Th1 type cytokines, IL-2 and IFNγ, compared with control [10]. Compared with thalidomide, lenalidomide is 50–2000 times more potent in inducing T-cell proliferation, and 300 to >1200 times more potent in augmenting T-cell IL-2 and IFN-γ production [9,10,11], and pomalidomide is the most potent of the three drugs [9,12].

It is suggested that the newer IMiDs inhibit IL-2-mediated generation of regulatory T cells. In a recent study, when a PBMC population was maintained in IL-2 over a period of 7 days, the addition of lenalidomide (IC_50_, 10 μM) or pomalidomide (IC_50_, 1 μM) significantly decreased the proportion/number of regulatory T cells and nuclear transcription factor Forkhead box P3 (FoxP3) in the population, compared with untreated controls [13]. It is thought that regulatory T cells suppression may promote tumor control but there are conflicting results showing some MM patients on IMiDs with increased regulatory T cells numbers despite tumor response [14,15]. A likely explanation is that the in vivo system is much more complex, and the cytokine milieu and tumor microenvironment play a yet unknown role in this system.

NK-T cells are T lymphocytes which bear NK cell surface markers with direct cytotoxic anti-tumor effect, produce IFN-γ and activate NK cells and DC [16]. The addition of lenalidomide potentiates the degree of DC-induced NK-T cell expansion and NK-T cell secretion of IFN-γ [17]. NK-T cell expansion, in turn, partially accounts for the activation and proliferation of NK cells associated with IMiDs [18] and perhaps also CD4 and CD8 T cells. NK cells have an important role in innate immunity, in killing both tumor and virus-infected cells.

Lenalidomide and pomalidomide (but not thalidomide) have been shown to enhance antibody-dependent cellular cytotoxicity (ADCC) and natural cytotoxicity of NK cells [18,19]. In the presence of lenalidomide and pomalidomide, there is increased NK cell Fas Ligand (FasL) and granzyme B expression leading to tumor cell apoptosis [20]. Through ADCC augmentation, IMiDs also enhance the cytotoxicity effects of monoclonal antibodies (see the next section). Overall, IMiDs stimulate T, NK-T and NK cells for tumor control. The newer IMiDs have the additional ability to cause tumor apoptosis via ADCC.

### 2.2. Clinical Studies

#### 2.2.1. Thalidomide

Thalidomide was the first IMiD used for treatment of MM patients, being the first new drug which showed single-agent activity for MM in more than three decades at that time [21]. It is still used in some countries, particularly in combination with bortezomib and dexamethasone as an induction therapy [22]. The CASSIOPEIA trial showed improved outcomes with daratumumab-bortezomib-thalidomide-dexamethasone combination, which we will discuss further in the daratumumab section. Some studies also showed improved outcomes with thalidomide maintenance therapy, however its use in clinical practice is restricted by its significant side effects [23].

#### 2.2.2. Lenalidomide

Lenalidomide is widely used in clinical practice as the main backbone of combination chemotherapy. The FIRST trial showed that lenalidomide-dexamethasone as a continuous treatment was superior to MPT (melphalan, prednisone, thalidomide) [24]. As compared to MPT, continuous lenalidomide-dexamethasone provided longer median progression-free survival (PFS) (25.5 months vs. 21.2 months; hazard ratio (HR), 0.72; *p* < 0.001) and higher 4 years overall survival (OS) rate (59% vs. 51%; HR, 0.78; 95% CI, 0.64 to 0.96; *p* = 0.02). The benefit of continuous lenalidomide-dexamethasone was seen in most subgroups, however, the benefit was questionable in patients with high-risk cytogenetics; HR 0.89 (95% CI, 0.56–1.43). There were less grade 3 or 4 adverse events for transplant-ineligible MM patients.

Lenalidomide is widely used as maintenance therapy, both after autologous stem cell transplantation (ASCT) and in transplant-ineligible patients. Encouraging data from large phase 3 trials has prompted the use of a triple combination of bortezomib, lenalidomide and dexamethasone as induction therapy for both transplant-eligible and ineligible patients [25,26,27]. Encouraged by the result of the MAIA trial, some centers are now using daratumumab plus lenalidomide and dexamethasone for transplant-ineligible MM patients [28], which we will discuss later in the daratumumab section.

Lenalidomide is also widely used as backbone of chemotherapy regimen in relapsed disease. In the POLLUX trial, daratumumab plus lenalidomide-dexamethasone prolonged PFS at 12 months (83.2% vs. 60.1% in the lenalidomide-dexamethasone group) [29]. Similarly, in the ASPIRE trial, carfilzomib plus lenalidomide-dexamethasone prolonged median PFS (26.3 months vs. 17.6 months in lenalidomide-dexamethasone group; HR, 0.69; *p* = 0.0001) [30].

Multiple randomized controlled trials have shown improved outcomes with lenalidomide maintenance therapy. The phase 3 study by the French Intergroupe Francophone du Myélome (IFM) showed improved median PFS (41 months vs. 23 months; *p* < 0 .001) in the lenalidomide maintenance group, as compared with the placebo group, although there was no OS benefit at 4 years [31]. Another study by the Cancer and Leukemia Group B (CALGB) showed both improved PFS (46 months vs. 27 months; *p*  <  0.001) and OS at 3 years (88% vs. 80%) [32]. The GIMEMA study showed significantly reduced risk of disease progression or death (41.9 months vs. 21.6 months; HR, 0.47; 95% CI, 0.33 to 0.65; *p* < 0.001) with lenalidomide maintenance therapy [33].

A meta-analysis evaluating the role on lenalidomide maintenance post-ASCT showed both PFS (median PFS was 52.8 months for the lenalidomide group and 23.5 months for the placebo or observation group; HR, 0.48) and OS (not reached for the lenalidomide maintenance group vs. 86.0 months for the placebo or observation group; HR, 0.75; *p* = 0.001) benefit [34]. However, the meta-analysis showed no OS benefit for patients with high-risk cytogenetics [34], for whom the benefit of lenalidomide maintenance therapy remains controversial. The recent Myeloma XI trial showed lenalidomide maintenance therapy improved PFS (median PFS 39 vs. 20 months; HR 0.46 [95% CI 0.41–0.53]; *p* < 0.0001), but with no OS benefit, with 3-year overall survival of 78.6% (95% Cl 75.6–81.6) in the lenalidomide group and 75.8% (72.4–79.2) in the observation group (HR 0.87 [95% CI 0.73–1.05]; *p* = 0.15) [35].

#### 2.2.3. Pomalidomide

With the widespread use of lenalidomide and increase in cases refractory to lenalidomide, pomalidomide has emerged as an important IMiD in this era. MM-002 [36] clinical trials confirmed the efficacy of pomalidomide and dexamethasone combination in patients who had previously been exposed to bortezomib and lenalidomide (median PFS of 4.2 vs. 2.7 months in pomalidomide only group; HR, 0.68; *p* = 0.003), leading to U.S. Food and Drug Administration (FDA) approval. The result was further confirmed by the MM-003 clinical trial (median PFS of 4.0 months; (95% CI 3.6–4.7) [37]. Interestingly, evaluation by the IFM group showed that pomalidomide and dexamethasone combination is active and well tolerated in early relapsed and/or refractory MM patients with adverse cytogenetics, particularly in those with del(17p) (Time to Progression (TTP), 7.3 vs. 2.8 months in patients with t (4;14)). Some studies have also shown that the addition of cyclophosphamide [38] or bortezomib [39] to this combination are effective.

## 3. Monoclonal Antibodies

In MM, several surface molecules have been explored as potential targets of monoclonal antibodies (MAbs). These include CD38, CD40, CD138, CD56, CD54, IL-6, PD1, CD74, CD162, b2-macroglobulin, kappa light chain, ganglioside GM-2, and the signaling lymphocyte activation molecule F7 (SLAMF7). To be considered for therapeutic use, these surface molecules selected by MAbs must have a high level of expression in MM cells and a low level of expression in normal cells.

In addition to targeting cell surface antigens, MAbs may modulate the non-cellular components of the bone marrow microenvironment, resulting in the neutralization of growth factors, inhibition of angiogenesis, modulation of mediators of bone disease, and enhancement of the host antitumor immune response. When used as monotherapy, MAbs generally do not produce a significant response in patients with myeloma, requiring it to be combined with other anti-myeloma agents for synergistic effects [40].

There are four main mechanism of action for MAbs: antibody-dependent cellular cytotoxicity (ADCC), antibody-dependent cellular phagocytosis (ADCP), complement-dependent cytotoxicity (CDC) and direct cell kill.

Therapeutic antibody-mediated ADCC results in lysis of antibody-coated tumor cells by effector cells. NK-cells play a critical role in ADCC mediated by therapeutic antibodies as NK-cell depletion markedly reduces daratumumab ADCC killing of MM cells [41]. Upon the binding of FcγRs (receptors for Fc portion of immunoglobulin) to the Fc tail of the CD38-targeting antibody, NK-cells release toxic proteins including granzymes and perforins, which will kill the target cells [42]. Macrophages, neutrophils, eosinophils, and γδ T-cells are able to induce ADCC against tumor cells coated with a therapeutic antibody but whether they play a role in CD38 antibody-induced ADCC is yet unknown [43].

In the process of ADCP, phagocytosis of antibody-opsonized tumor cells occurs via binding of Fcγ (such as FcγRIIA and FcγRIIIA), which are present on monocytes and macrophages. Phagocytosis is an efficient killing mechanism of daratumumab [44,45] where macrophages have the ability to quickly and sequentially engulf multiple daratumumab-coated tumor cells [45]. Uptake of antibody-opsonized cancer cells by APCs, such as macrophages and DCs may also lead to enhanced antigen presentation, which may contribute to the development of tumor antigen-specific CD4+ and CD8+ T-cell immune responses [46,47].

Binding of complement component C1q to the Fc tail of the therapeutic antibody initiates the complement cascade, ultimately resulting in the generation of the membrane attack complex (MAC) and subsequently permeabilization of the cell membrane [48,49]. C3-convertase cleaves and activates component C3, creating C3a and C3b, and causes a cascade of further cleavage and activation events. Deposition of C3b on the surface of the target cell results in the engulfment of the tumor cells by phagocytosis. In addition, complement activation also leads to the generation of C3a and C5a. C5a increases expression of activating FcγRs, while at the same time reduces inhibitory FcγRs, which leads to enhanced phagocytosis capacity of effector cells. C3a and C5a also recruit immune cells to the tumor. Altogether, this indicates that complement and the FcγR system act synergistically to eliminate tumor cells [50,51].

### 3.1. Anti-CD38

#### 3.1.1. Rationale and Mechanism

CD38 is a transmembrane receptor protein highly expressed on malignant plasma cells and on normal B cells during different stages of their maturation [52]. Under normal conditions, CD38 is expressed at relatively low levels on myeloid and lymphoid cells and in some non-hematopoietic tissues [53]. CD38 plays a role in regulation of migration, receptor-mediated adhesion by interaction with CD31 or hyaluronic acid, and signaling events [52,53,54]. CD38 MAbs currently available or being developed for the treatment of MM include daratumumab, isatuximab, and MOR202.

More recently, Krejcik and colleagues [55,56] have shown that daratumumab depletes CD38+ immune regulatory cells, and promoted the increase of T-helper cells, cytotoxic T cells, T-cell functional response, and TCR clonality. This finding suggested that drugs able to enhance the immune system may be preferred partners to combine with anti-CD38 MAbs.

IMiDs such as lenalidomide and pomalidomide induce NK-cell activation and synergize with daratumumab in ADCC assays [41,57]. Even in patients who are lenalidomide-refractory, addition of daratumumab to lenalidomide elicits clinical response. The addition of proteasome inhibitors enhances the effect of daratumumab by sensitizing tumor cells to ADCC, but the full mechanism has not been elucidated.

There is a rapid decrease in CD38 expression levels on the MM cell surface during daratumumab treatment [56,58]. Directly following the first daratumumab infusion, an approximately 90% reduction in CD38 expression levels is noticed on non-depleted MM cells (68). The reduction in CD38 cell surface expression is a transient phenomenon, because CD38 levels are restored to baseline levels on the MM cells approximately 6 months after the last daratumumab infusion [56]. Reduced CD38 expression may result in impaired adhesion to stromal cells via CD38–CD31 interactions, leading to reduced growth and impaired protection against apoptosis. This reduction in CD38 expression on MM cells does not occur with isatuximab.

Compensatory upregulation of multiple inhibitory immune checkpoints, which is implicated in the resistance to PD-1 or PD-L1 inhibitors, may also contribute to the development of resistance to the immunomodulatory activities of CD38 antibodies [59]. Indeed, pre-clinical data suggest that immunomodulatory activity of CD38 antibodies can be enhanced by combining a CD38 antibody with a PD-1/PD-L1 inhibitor. For example, in mouse models targeting the CD38 and PD-1 pathway in MM, lung cancer, and colon adenocarcinoma, the combination of a CD38 antibody and PD-1 antibody resulted in enhanced anti-tumor activity, when compared to targeting either pathway alone [60]. This was accompanied by increased T-cell infiltration and T-cell activation in the tumors with combined anti-CD38 and anti-PD-1 treatment [60]. In addition, another group showed that CD38 expression is increased following therapy with a PD-L1 inhibitor in a lung cancer mouse model, which was associated with impaired CD8+ T-cell function, and the combination of a CD38 antibody with PD-L1 inhibitor showed enhanced anti-tumor activity [61].

#### 3.1.2. Clinical Studies

In a pooled analysis of 148 patients who received daratumumab treatment as single agent at a dose of 16 mg/kg in the first-in-human phase 1/2 GEN501 study [62] and phase 2 SIRIUS study [63], at least partial response (PR) was achieved in 31% of the patients, including very good partial response (VGPR) or better in 18%. These patients were heavily pretreated with a median of five prior lines of therapy with 86% double-refractory to a proteasome inhibitor and an IMiD [43]. The median duration of response was 7.6 months (95% confidence interval [CI], 5.6 to not evaluable [NE]). The median PFS and median OS were 4.0 months (95% CI, 2.8–5.6 months) and 20.1 months (95% CI, 16.6 months to NE), respectively [64].

Common adverse events associated with single-agent daratumumab included fatigue (39.6%), anemia (33%), nausea (29.2%), and thrombocytopenia (25.5%) [62,63]. Infusional events, which were mostly of grade 1 or 2, were seen in 42.5% of patients, mainly during the first infusion [62,63]. Most patients tolerated the infusion and the incidence of infusional events reduced with further therapy. Premedication with corticosteroid and a longer infusion time reduced infusional reactions.

Both Phase 3 trials comparing the combination of daratumumab with bortezomib, and dexamethasone (CASTOR) or lenalidomide and dexamethasone (POLLUX), versus the drugs alone in relapsed or refractory myeloma demonstrated improved PFS; CASTOR: 16.7 vs. 7.1 months; HR, 0.31, 95% confidence interval (CI) 0.24–0.39, *p* < 0.001; POLLUX: not reached vs. 17.5 months; HR, 0.41, 95% CI, 0.31–0.51, *p* < 0.0001). There was also increased overall response with both trials compared to daratumumab alone. This increase in response came at the cost of increased hematologic toxicities [29,65,66,67] but no increased infusional reactions.

FDA recently approved daratumumab combination regimen to be used as induction therapy for newly-diagnosed transplant-eligible patients based on the results of the randomized phase 3 CASSIOPEIA study which showed that daratumumab with bortezomib, thalidomide, and dexamethasone (D-VTd) improved depth of response and PFS as compared to VTd alone (stringent complete response (sCRs): 29% vs. 20% (odds ratio 1.60, 95% CI 1.21–2.12, *p* = 0.0010)); complete response (CR) or better: 39% vs. 26%, and minimal residual disease (MRD) negativity: 64% vs. 44% (10^−5^ sensitivity threshold, assessed by multiparametric flow cytometry; both *p* < 0.0001) [68]. This approval followed the earlier approval of daratumumab use as induction therapy for newly-diagnosed transplant-ineligible MM patients following the result of the phase 3 MAIA study, which showed that daratumumab with lenalidomide-dexamethasone combination significantly reduced the risk of disease progression or death compared to treatment with lenalidomide-dexamethasone alone. The estimated percentage of patients who were alive without disease progression at 30 months was 70.6% (95% confidence interval [CI], 65.0 to 75.4) in the daratumumab group as compared with 55.6% (95% CI, 49.5 to 61.3) in the control group (HR, 0.56; 95% CI, 0.43 to 0.73; *p* < 0.001) [28]. The ALCYONE trial by the Spanish has earlier shown that among patients with newly diagnosed MM ineligible for stem-cell transplantation, daratumumab combined with bortezomib, melphalan, and prednisone resulted in a lower risk of disease progression or death than the same regimen without daratumumab (18-month PFS was 71.6% vs. 50.2%; HR, 0.50; 95% CI, 0.38 to 0.65; *p* < 0.001) [69]. It will be informative when the final results of the GRIFFIN trial is published as this trial evaluates transplant-eligible newly diagnosed MM patients treated with daratumumab in combination with bortezomib, lenalidomide and dexamethasone.

The quadruplet combination of daratumumab-carfilzomib, lenalidomide and dexamethasone (Dara-KRD) is currently being evaluated in a clinical trial and early result has shown that it is safe and leads to high proportion of patients achieving CR/sCR, MRD-negative CR, and imaging plus MRD-negative CR [70].

A phase 3 study of isatuximab, another monoclonal antibody targeting CD38, plus pomalidomide and dexamethasone versus pomalidomide and dexamethasone alone in relapsed/refractory multiple myeloma, the ICARIA-MM Phase III study, has also just been published. At a median follow-up of 11.6 months, the median progression-free survival was 11.5 months in the isatuximab-pomalidomide-dexamethasone group versus 6.5 months in the pomalidomide-dexamethasone group (HR 0.596, 95% CI 0.44–0.81; *p* = 0.001). One important caveat in this trial is that patients who were refractory to previous treatment with an anti-CD38 monoclonal antibody were excluded [71].

### 3.2. Anti-SLAMF7

#### 3.2.1. Rationale and Mechanism

SLAMF7 (also known as CS1 or CD319) stands for family member 7 (F7) of the signalling lymphocytic activation molecule (SLAM) family, a subset of the immunoglobulin superfamily of receptors expressed on several hematopoietic cells, including myeloma cells [72]. The SLAM family consists of transmembrane proteins that share a similar motif of an extracellular domain, a transmembrane domain, and a cytoplasmic domain. Together, the SLAM family members are immunomodulatory receptors with roles in cytotoxicity, humoral immunity, autoimmunity, cell survival, lymphocyte development, and cell adhesion [72].

As a key regulator of normal immune cell function, SLAMF7 activates NK cells and is thought to have a growth-promoting role in normal B-cell development and an inhibitory role in T-cell development. It may possess a tumor-stimulating effect by promoting myeloma cell adhesion to bone marrow stromal cells in the microenvironment [73,74].

The high level of SLAMF7 expression on myeloma cells prompted the development of elotuzumab, a humanized MAb that binds to SLAMF7. Elotuzumab received regulatory approval for the treatment of relapsed or refractory myeloma in combination with lenalidomide and dexamethasone in patients who have received one to three prior therapies. Elotuzumab has no direct cytotoxic or cytostatic effect on myeloma cells. It exerts its effect via a dual mechanism of action: direct activation of NK cells and ADCC via SLAMF7 ligation in which the Fc portion of elotuzumab binds to the activating Fc receptor (CD16) on NK cells, whereas the Fab portion of elotuzumab binds to SLAMF7 on myeloma cells [74,75]. As a result, NK cells that secrete cytokines such as interferon-γ are activated, further enhancing the anti-myeloma effect. Elotuzumab interferes with the function of the immunosuppressive cells in the microenvironment and augments the activity of other effector cells [74].

#### 3.2.2. Clinical Studies

A phase 1 dose escalation trial of 34 heavily pretreated patients revealed a favorable toxicity profile but disappointingly, there were no objective responses, and stable disease was reported in 26% of patients [76]. However, with the addition of lenalidomide, in a phase 3 trial comparing elotuzumab, lenalidomide and dexamethasone vs. lenalidomide and dexamethasone, the triplet showed an ORR of 79% vs. 66% and PFS of 41% vs. 27%, respectively (recently updated 5-year follow up data) [77,78].

The recently published Eloquent-3 trial with elotuzumab plus pomalidomide and dexamethasone for relapsed/refractory MM as compared with pomalidomide and dexamethasone alone showed improved PFS of 10.3 vs. 4.7 months (HR of 0.54; 95% CI, 0.34 to 0.86; *p* = 0.008) in the elotuzumab group [79].

### 3.3. Other Monoclonal Antibodies 

IL-6 is a cytokine that has been implicated in the proliferation and survival of MM cells. Pre-clinical studies suggested that the combination of siltuximab (an anti–IL-6 monoclonal antibody) and bortezomib might have synergistic effects. However, the results of a randomized control trial in combination with bortezomib failed to report statistically significant differences in response rate, PFS, or OS, whereas it did increase the frequency of adverse events, including cytopenias [80,81]. Currently, it is being tested in patients with high-risk smoldering myeloma. Other antibodies being tested include anti-B-cell maturation antigen (BCMA) and tabalumab, a human anti-B-cell activating factor antibody [82]. The monoclonal antibodies are listed in Table 1.

## 4. Antibody-Drug Conjugate

### 4.1. Rationale and Mechanism

Antibody–drug conjugate (ADC) is a recombinant MAb conjugated to a small-molecule cytotoxin. ADC targets antigens on myeloma cells and delivers the cytotoxic agent directly to the myeloma cells following internalisation of the compounds after binding to the target antigen [108]. It has received great attention recently due to its ‘off-the-shelf’ availability, as compared with chimeric antigen receptor (CAR) T-cell therapy.

BCMA, also known as CD269, is a member of the TNF receptor superfamily that is predominantly expressed on normal and malignant plasma cells, along with a small subset of mature B cells and plasmacytoid DCs; it is not expressed in hematopoietic stem cells or nonhematologic cells [109]. This makes BCMA a good target for immunotherapies.

### 4.2. Clinical Studies

A few ADCs targeting different myeloma antigens have been developed. Belantamab mafodotin (GSK2857916), targeting BCMA, has shown promising results in a phase 1 study. The updated result of the dose expansion phase I study of the antibody–drug conjugate against BCMA, belantamab mafodotin, showed median progression-free survival was 12.0 months (95% CI 3.1–not estimable) and the median duration of response was 14.3 months (95% CI 10.6–NE) in relapsed and/or refractory MM patients. The ORR was 71% in those without prior daratumumab [110], and 42.9% with a PFS of 6.8 months in patients refractory to daratumumab [110]. Although the response was worse in the daratumumab-refractory cohort, the result was still better than the 31% ORR seen in the evaluation for the other first treatment regimen after progression with anti-CD38 therapy, with a median PFS of just 3.4 months [111]. The recently published phase 2 DREAMM-2 study showed that 30 (31%; 97.5% CI 20.8–42.6) of 97 patients in the 2.5 mg/kg cohort and 34 (34%; 23.9–46.0) of 99 patients in the 3.4 mg/kg cohort achieved an overall response [112].

Indatuximab ravtansine (BT062), an anti-CD138 monoclonal antibody conjugated with drug maytansinoid 4 (DM4), a cytotoxic maytansinoid derivative [113] has been shown to possess a favorable safety profile, with nausea, anemia, diarrhea, and fatigue as the most common adverse events in a dose-escalating phase 1 trial of 29 patients with relapsed and/or refractory MM [102]. Most patients had stable disease when treated as monotherapy [101], however when combined with lenalidomide, the ORR improved to 78% [103].

Lorvotuzumab mertansine is a humanized anti-CD56 monoclonal antibody conjugated to drug maytansinoid 1 (DM1), another cytotoxic maytansinoid derivative. CD56 is expressed on MM cells and NK cells and neural tissue. Phase 1 monotherapy trials in CD56-positive relapsed and/or refractory MM showed an ORR of 7%. The toxicity profile was acceptable, consisting mostly of peripheral neuropathy, cytopenias, and fatigue [114]. Combination therapy with lenalidomide and dexamethasone increased the ORR to 56% [106]. ADCs that target CD74 (milatuzumab-doxorubicin) and FcRH5 (DFRF4539A) are also being investigated in early phase clinical trials.

## 5. Bi-Specific T-Cell Engagers

### 5.1. Rationale and Mechanism

Bi-specific T-cell engagers (BiTE) are an innovative class of immunotherapy which brings tumor cells into contact with T-cells [115]. BiTE antibodies bind to their target tumor cell as well as T-cells, bringing the two into physical proximity [115]. This leads to the formation of a cytolytic synapse allowing the T-cells to release cytotoxic molecules leading to tumor cell death [115]. The CD19 BiTE blinatumomab has proven to be a potent therapeutic option in relapsed B acute lymphoblastic leukemia [116].

BiTE therapy in MM is an area of active research [115]. The identification of cell surface antigens that are specifically expressed on the tumor cell and not normal tissues is a requirement for successful application of BITE therapy [115]. Several target antigens for BiTE have been explored in MM; CD 138, target of wue-1 antibody and Fc receptor like 5 (Fcrl 5) being examples [115]. Among the targets studied so far, the BCMA is considered one of the most promising [115]. BCMA is highly specific to plasma cells and is expressed at higher intensity in MM compared to monoclonal gammopathy of undetermined significance (MGUS) and in normal individuals. It has therefore been actively investigated as a target for BiTE in MM [117]. AMG 420, a BCMA BiTE has been shown to induce selective lysis of BCMA positive MM cell lines as well as patient samples [118]. Impressive responses were also seen in animal models suggesting that BCMA BiTE therapy has potent in vivo activity [118]. Similar impressive data were reported for other BCMA BiTEs (EM801 and BiFab-BCMA) [119,120]. These agents also induced lysis of MM cells from high-risk patients, as well as tumor regression in murine models.

### 5.2. Clinical Studies

AMG424, a BiTE targeting CD38, has also been reported to show potent activity against MM cell lines, with both low and high CD38 expression in vitro [121]. This agent also inhibited tumor growth in murine models and showed acceptable toxicity in monkeys. The expression of CD38 on NK cells, monocytes and a subset of B cells raises the question of the impact of this therapy on other immune effector cells [121,122]. Zuch de Zafra et al. showed that although therapeutic doses of AMG424 resulted in B cell depletion, significantly higher doses were required to affect NK cell, monocytes and T-cells [121].

Based on these promising data, early phase clinical trials investigating BiTEs in MM are in progress. AMG 420 is an anti-BCMA BiTE immunotherapy agent which binds BCMA on MM cells and CD3 on T cells. The first-in-human study of 42 patients receiving AMG 420 showed that the response rate was 70% in relapsed and/or refractory MM patients, including 50% MRD-negative complete responses, at 400 μg/d, the MTD for this study [123].

Several questions however remain to be answered. The durability of responses seen with BiTEs will require longer follow up of patients in larger studies. The question of whether CD38 BiTEs would still be effective in patients previously treated with daratumumab would be of importance. As with other novel therapies, key areas to be addressed in future studies are: (i) which patient population is most likely to benefit; and (ii) at what stage of disease should the treatment be used. Whether BiTE can be combined or sequenced with existing MM therapies would also be an important area for investigation. 

## 6. Immune Checkpoint Inhibitors

### 6.1. Rationale and Mechanism

Immune checkpoints have evolved to protect organisms from T-cell-mediated autoimmunity [124]. Malignancies have exploited these pathways to evade the host T-cell immune response, hence escape from immune surveillance is recognized as a hallmark of cancer [125]. Examples of immune checkpoints relevant to oncology include CTLA4, LAG3, TIM3, TGIT and programmed death receptor 1 (PD-1) [126]. The PD-1/PD-1 ligand (PDL-1) pathway is critical to the normal regulation of T-cell mediated immunity [127]. PD-1 is expressed on normal T-cells while PDL-1 is expressed on antigen presenting cells as well as a number of other tissues [127]. Binding of PD-1 to its ligand results in inhibition of T-cell mediated cytotoxicity [127]. Examples of tumors over expressing PDL-1 include melanoma, lung adenocarcinoma and Hodgkin lymphoma (HL) [128]. Therapeutic targeting of the PD-1/PD-L1 pathway has revolutionized the treatment of these cancers and is under active investigation in other hematologic malignancies, including MM [128].

There is also data suggesting that the PD-1/PD-L1 pathway may not be the only determinant of immune dysregulation in MM. It has been proposed that T-cell senescence may play a more prominent role than increased PD-1 expression [129]. PD-L1 expression on solid tumors is related to mutational burden and neo antigen expression [130]. MM has a relatively low mutational burden compared to many solid tumors and hence has a different profile of neo antigen expression [131]. Furthermore, the sensitivity to checkpoint inhibition is more pronounced in tumors with high immune cell infiltration as is seen in melanoma and HL [131]. MM, however, is not characterized by significant immune cell infiltrate. Taken together, these data suggest that although checkpoint inhibitors may have potential as a therapeutic option in MM, they may not be as potent against MM as they are against solid tumors.

### 6.2. Clinical Studies

A phase 1b study of single agent nivolumab, a PD-1 inhibitor, in 27 relapsed refractory MM patients showed a best response of stable disease, suggesting that single agent activity is limited [132]. Similarly, the single agent activity of the PD-1 inhibitor pembrolizumab was modest in the relapsed refractory setting, as well as when used as consolidation post-ASCT [126,133]. Lenalidomide has been shown to down regulate PD-1 expression on T and NK cells while reducing expression of PD-L1 in MM cells in vitro [134]. In combination with PD-1 inhibitors, lenalidomide was shown to up-regulate interferon gamma (IFN-γ) secretion by bone marrow effector cells and induce apoptosis of MM cells in vitro [135]. Based on these data, several clinical trials are currently evaluating the efficacy and safety of PD-1 inhibition in combination with IMiD therapy.

The combination of pembrolizumab, lenalidomide and dexamethasone in a phase 1 study showed an ORR of 76% in relapsed/refractory MM [136]. Phase 3 studies evaluating pembrolizumab in combination with lenalidomide and dexamethasone (KEYNOTE-185) and pomalidomide and dexamethasone (KEYNOTE-183), respectively. were initiated following these encouraging data [136,137]. Unexpectedly, both trials showed a higher mortality in the pembrolizumab arms with no significant difference in terms of disease response. This led to the FDA temporarily halting recruitment to several clinical trials of anti PD-1/PD-L1 therapy in MM [126]. It is noteworthy that the median age, incidence of renal impairment and high risk cytogenetics was higher in the pembrolizumab arm of the KEYNOTE-185 trial [137]. Although these factors may partly account for the discrepant mortality, further work is required to fully explain the results from both trials.

Following review of safety data, several trials investigating anti PD-1 therapy in MM have since recommenced recruitment. A phase 1 trial of nivolumab in combination with IMiD, daratumumab and proteasome inhibitors has shown an acceptable toxicity profile [138]. Data on anti PDL-1 (atezolizumab and durvalumab) agents in combination with existing myeloma therapies are eagerly awaited.

It is noteworthy that many of the clinical trials investigating anti PD-1 therapy in MM used them in combination with steroids [126,130]. It is well known that steroids affect T-cell mediated immunity and have been proposed to adversely affect immune checkpoint inhibitor therapy [139]. It is hence interesting to speculate whether the use of steroids together with anti PD-1 therapy had a negative impact on efficacy in the trials on MM patients.

Taken together, these data suggest that a better understanding of the immune profile of MM is required before we can harness the full potential of checkpoint inhibitors in this disease. Further studies are required to better understand the role of the immune system in disease progression from MGUS to MM, as well as in maintaining remission status post-treatment. How best to combine checkpoint inhibitors with existing myeloma therapies is another aspect which requires careful consideration. Pre-clinical data supporting the use of checkpoint inhibitors with monoclonal antibodies and even radiotherapy may provide avenues to design novel therapeutic regimens [60,140].

The identification of which MM patients are most likely to benefit from checkpoint inhibition as well as what is the optimal timing to introduce these agents are important questions to be addressed in future trials. Finally, the combination of anti PD-1 therapy with other T-cell stimulatory agents such as CTLA4 inhibitors or tumor vaccines are exciting areas for future research [131].

## 7. Chimeric Antigen Receptor (CAR) T-Cell Therapies (CAR-T)

### 7.1. Rationale and Mechanism

Chimeric antigen receptor (CAR) T-cells (CAR-T) therapy is a form of adoptive T-cell therapy in which T cells collected from a patient are genetically modified to express chimeric antigen receptors (CARs), which are artificial fusion proteins that incorporate an antigen-recognition domain and T-cell signaling domains [141,142,143]. T cells expressing a CAR can specifically recognize a targeted antigen, which is an advantage of CAR-T cells over nonspecific cellular therapies such as allogeneic hematopoietic stem cell transplantation [141,142,143]. CARs are not HLA-restricted, so patients of any HLA type can be treated with CAR-T cells; this is an advantage of CAR-T cells over T cells engineered to express HLA-restricted TCRs [141,144,145]. In addition to an antigen-recognition domain, CARs include hinge and transmembrane regions that connect the extracellular antigen-recognition domain to cytoplasmic signaling domains [141,142,143]. Inclusion of co-stimulatory domains in CARs has been a critical step in the development of CAR-T therapies [141,146,147,148,149]. From first-generation CARs which contained CD3ζ, which was sufficient to activate a weak T-cell response [142,144,150], we now have second-generation CARs, which added a second co-stimulatory signal, which could be a CD28, 4-1BB (CD137), OX40 (CD134), or an immune T-cell co-stimulator, leading to more robust cytokine production and enhanced cytolytic capacity of CAR-T cells [141,146,147,148,149].

A key factor in the development of a successful CAR, similar to monoclonal antibodies, is choosing an appropriate surface antigen target that is absent in normal cells but abundantly present in tumor cells. Currently, multiple antigen targets, including BCMA, CD19, kappa light chain, CD38, CD138, and SLAMF7, are being actively studied in clinical trials.

### 7.2. Clinical Studies

The US National Cancer Institute (NCI) conducted the first-in-humans clinical trial of CAR-BCMA in heavily treated relapsed and/or refractory MM patients. The CAR-BCMA construct contained a murine anti-BCMA single-chain variable fragment, hinge and transmembrane regions from human CD8α, the CD28 costimulatory domain, and the CD3ζ T-cell activation domain. Of the 16 patients who received 9 × 10^6^ CAR-BCMA T cells/kg at the highest dose level of the trial, the ORR was 81%, with 63% very good partial response or complete response. All 11 patients with partial response or better and had MM evaluable for minimal residual disease achieved bone marrow minimal residual disease—negativity. Although some had severe cytokine-release syndrome (CRS) toxicities, they were reversible [151].

A phase 1, open-label study from Nanjing Legend Biotech in China (LCAR-B38M) has a dual epitope-binding against 2 distinct BCMA epitopes, with a 4-1BB co-stimulatory domain and lentiviral vector used for transduction. Of the 57 patients treated with CAR T cell infusions at doses of 0.07 to 2.1 × 10^6^ cells/kg (median 0.5 × 10^6^ cells/kg), ORR was 88%, with 68% achieved a complete response, 5% achieved a very good partial response, and 14% achieved a partial response. Minimal residual disease was negative for 63% patients. CRS occurred in 90% of patients, with 7% having grade ≥ 3 cases. One patient reported neurotoxicity of grade 1 aphasia, agitation, and seizure-like activity [152].

The recently published phase 1 of bb2121 CAR-T cell therapy targeting BCMA for relapsed and/or refractory MM with at least 3 prior lines of therapies showed ORR of 85%, including 45% patients with complete responses [153]. From the 15 patients who had complete response, 6 had relapsed. The median progression-free survival was 11.8 months (95% CI, 6.2 to 17.8). All 16 patients who had a PR or better and who could be evaluated for MRD had MRD-negative status (≤10^−4^ nucleated cells) [153]. Of particular interest, it also showed good efficacy with response rate of 73% in patients with adverse cytogenetic profile, albeit lower as compared to the entire cohort [153].

The KarMMa-3 randomized phase 3 trial evaluating the efficacy and safety of bb2121 vs. standard triplet regimens in patients with relapsed and refractory MM is currently ongoing. Another exciting result is the interim result of the CARTITUDE-1 trial, a phase 1b/2 study of JNJ-4528, a CAR-T cell therapy containing two BCMA-targeting single-domain antibodies in relapsed and/or refractory MM, which demonstrated that at a target dose of 0.75 × 10^6^ CAR+ cells/kg, it could deliver ORR of 91% with early and deep response, with a manageable safety profile [154].

Though CD19 is not expressed on most MM cells, minor CD19+ subsets of the myeloma clone may harbor unique disease-propagating capabilities and may play a role in disease relapse. Garfall et al. evaluated CAR against CD19 (CTL019) following salvage high-dose melphalan and ASCT, in relapse and/or refractory MM patients. The study showed that 2 of 10 patients had significantly longer PFS after ASCT + CTL019 compared with prior ASCT (479 vs. 181 days; 249 vs. 127 days) [155]. 

Besides the trials described here, several CAR trials exploring other targets for the treatment of MM are currently ongoing (Table 2).

## 8. Oncolytic Virotherapies

### 8.1. Rationale and Mechanism

Oncolytic virus (OV) therapy leads to tumor cell death via two distinct mechanisms: direct oncolytics or oncolytic immunotherapy [175,176]. Direct oncolytics involves the virus directly killing the tumor cell via lytic viral replication or induction of cell death [176]. In oncolytic immunotherapy, viral infection results in activation of the host’s anti-tumor immune response [175]. Several naturally occurring viruses have been used as agents for OV therapy, with modifications to enhance their specificity for tumor cells. A number of characteristics make MM an attractive target for OV [177]. Overexpression of specific cell surface receptors allows some viruses easier access to MM cells while altered IFN-γ and protein kinase R (PKR) activity render MM cells more suitable for viral replication [177,178]. There are a number of OV agents currently in development.

#### 8.1.1. Measles Virus

The measles virus (MV) is one of the best studied agents for OV in hematologic malignancies [177]. MM cells significantly overexpress the MV receptor CD46 compared to normal hematopoietic cells, making them more susceptible to infection by MV [179]. The mechanism behind MV-mediated MM cell death is not fully understood. Lytic viral replication is likely to be a major mechanism while formation of syncitia may also contribute [177]. MV therapy has shown significant efficacy in patient-derived xenograft ( PDX) models of MM [180]. The study of MV in these models has however been limited by the fact that MV does not naturally infect mice [181]. Furthermore, antibodies against MV are passively transferred to the mice when human MM cells are delivered via tail vein injection, resulting in some neutralization of MV in the PDX [182]. The use of immunodeficient PDX models also hampers the study of oncolytic immunotherapy induced by MV [177]. Despite these hurdles, MV has progressed into clinical trials for relapsed/refractory MM. A phase 1 study showed 4 of 11 patients achieving a response with one complete remission, albeit with significant hematologic toxicity [183]. Most patients in this study developed antibodies against MV, highlighting another potential obstacle to clinical application of MV therapy. MV nevertheless remains the most advanced and promising of the OV agents against MM and future studies should aim to overcome the barriers to its clinical progress.

#### 8.1.2. Reovirus

Reovirus is a promising choice for OV in MM due to the expression of the Reovirus receptor JAM-A on malignant plasma cells [184]. This is supported by the finding that MM cells with higher JAM-A expression are more sensitive to reovirus treatment [184]. Lytic viral replication is likely to be the dominant mechanism of reovirus-mediated MM cell death, however induction of apoptosis and autophagy has also been proposed [185]. Interestingly, reovirus was also shown to affect the unfolded protein response which is crucial for the survival of MM cells [186]. The efficacy of reovirus in pre-clinical models has been modest and a phase 1 clinical trial showed stable disease as the best response achieved [185,187]. These data suggest that reovirus maybe more effective as part of combination therapy, in particular with agents upregulating JAM-A expression on MM cells [188]. The ability of reovirus to selectively infect MM cells found at low levels in mixtures of normal bone marrow aspirates has led to the proposal of it being used as a purging agent for stem cell harvest prior to ASCT [189]. Further studies are required to define the optimal role of this agent in MM OV therapy.

#### 8.1.3. Adenovirus

The serotype of adenovirus employed for OV appears to be an important determinant of efficacy against MM. Serotypes 6, 26 and 48 were found to have anti-MM activity, while serotypes 11, 35, 40 and 41 were toxic to PBMCs but not MM cells [190]. The explanation for the differential efficacy between serotypes maybe more related to replication kinetics than viral adsorption or receptor upregulation as described for other viruses [177]. The in vivo efficacy of adenovirus therapy has been modest, leading to attempts at modifying the virus to enhance its killing capability [177,190]. An important barrier to the clinical application of adenovirus in OV therapy is host antibodies. Most patients would have been exposed to this pathogen at some point in their life and are likely to have humoral immunity against it [177]. Identifying the best serotype of adenovirus to target MM and optimizing the efficacy while overcoming host immunity are challenges to be addressed in future studies.

#### 8.1.4. Vesicular Stomatitis Virus

Vesicular stomatitis virus (VSV) is a potent OV agent which kills MM cells by lytic replication as well as inhibition of DNA and RNA synthesis [177,191]. Interestingly, oncolytic immunotherapy is not a mechanism of VSV-mediated MM cell death; indeed, VSV OV was more effective in immunodeficient compared to immune-competent mouse models of MM [192,193]. It is noteworthy that VSV was found to infect neutrophils and monocytes in the peripheral blood of mice [194]. Infective virus was also isolated from the spleen of treated animals and systemic inflammatory responses as well as hepatic toxicity were reported [195]. Fatal central nervous system toxicity was reported in mice treated with VSV and had meningeal MM deposits [193]. Despite its potency against MM, the concerns of serious toxicity must be evaluated carefully prior to further clinical development of VSV as an OV agent against MM.

#### 8.1.5. Vaccinia Virus

The vaccinia virus has shown promising results in the treatment of solid tumors but data in hematologic malignancies is less robust [177]. In MM, the evidence for vaccinia as an OV agent are conflicting, with some studies showing potent lytic activity while others showing only modest reductions in cell viability [196,197]. Given the historical data supporting the use of this virus as a base for vaccines, ongoing studies are aiming to establish whether vaccinia can be used to generate anti-myeloma vaccines [177].

#### 8.1.6. Other Viruses 

A number of other viruses are currently being investigated as potential OV agents. Myxoma virus causes disease in rabbits and is not pathogenic in humans [177]. Unlike the other agents discussed so far, myxoma virus induces cell death in MM cells by induction of extrinsic apoptosis rather than lytic replication [177,198]. This agent has shown potential as a purging agent for stem cell harvests due to its ability to selectively infect plasma cells [199].

Coxsackie virus causes mild respiratory disease in humans [200]. It has shown promising pre-clinical activity in cell lines and murine models [201]. Clinical application of this agent may be hampered by its potential to cause myositis in humans, but this may be attenuated by the incorporation of muscle-specific microRNA into the viral genome [202].

### 8.2. Clinical Studies

The development of host immunity against viruses is an important barrier to clinical application of OV [177,200]. Suppressing the host immune response with cyclophosphamide has been proposed as a means to overcome this hurdle [203]. As cyclophosphamide is commonly used in MM treatment protocols, it is an attractive option for combination therapy [200]. Combining IMiDs with OV may also be a promising option for future clinical trials given that OV augments the immune response and may potentially enhance the activity of IMiDs [200]. Given the evidence that reovirus disrupts the unfolded protein response, combination of this agent with proteasome inhibitors is an attractive option for further studies. Phase 1 clinical trials are currently evaluating this combination in relapsed and/or refractory MM (NCT02514382) [200].

As discussed above, clinical trials of checkpoint inhibition have not been as successful in MM as they have been in solid tumors and lymphoma [131]. The use of OV to upregulate PDL-1 expression on MM cells has been demonstrated in vitro [200]. Indeed, reovirus treatment in combination with checkpoint inhibition proved an effective strategy in a murine model [200]. Combination with OV may therefore represent a means to sensitize MM cells to immune checkpoint inhibition and should be explored in future studies.

## 9. Vaccines

Cancer vaccines aim to stimulate the host immune response against the tumor predominantly by enhancing tumor antigen presentation [204]. The choice of tumor antigen to be used and the method by which antigen presentation is enhanced are key considerations in the design of cancer vaccines [205].

Vaccines have shown promising results in solid tumors, however their application in hematologic malignancies has been limited [205]. DCs are highly effective antigen presenting cells which have been extensively studied in the context of cancer vaccine therapy [206]. Physical fusion of DCs with myeloma cells (myeloma/DC fusion vaccine) was successfully employed in a phase 2 clinical trial where the vaccine was administered post-ASCT [207]. Fusion vaccines are also being evaluated in combination with checkpoint inhibitors and immunomodulators [205,208] Electroporation of MM-specific mRNA into Langerhans type DCs is currently under evaluation as an alternative DC-based vaccine therapy. This strategy is based on pre-clinical data suggesting that this subtype of DC induces more potent immune responses [205,209].

MM-derived proteins and peptides have also been evaluated as targets for vaccines in MM. The MAGE-A3 cancer testis antigen for example was used in combination with an autologous T-cell infusion post stem cell transplant resulting in antigen-specific T-cell responses [210]. Other MM specific antigens being studied in this context include XBP1, CS1 and CD138 and “idiotype” immunoglobulins unique to the MM clone [205].

**Figure 1:**
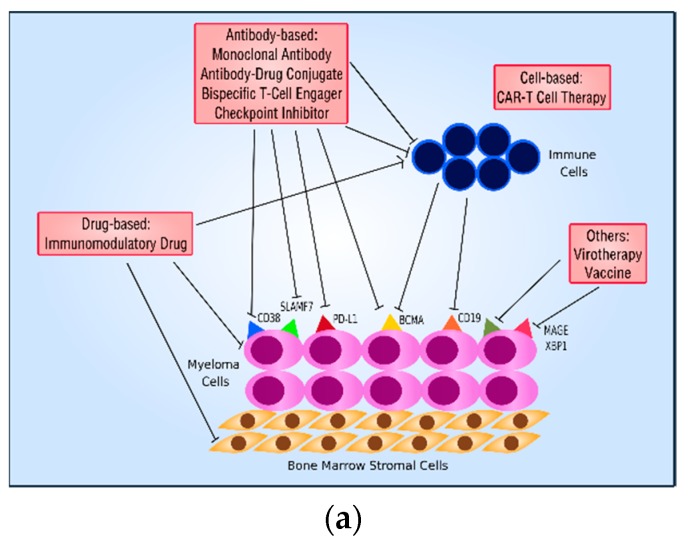

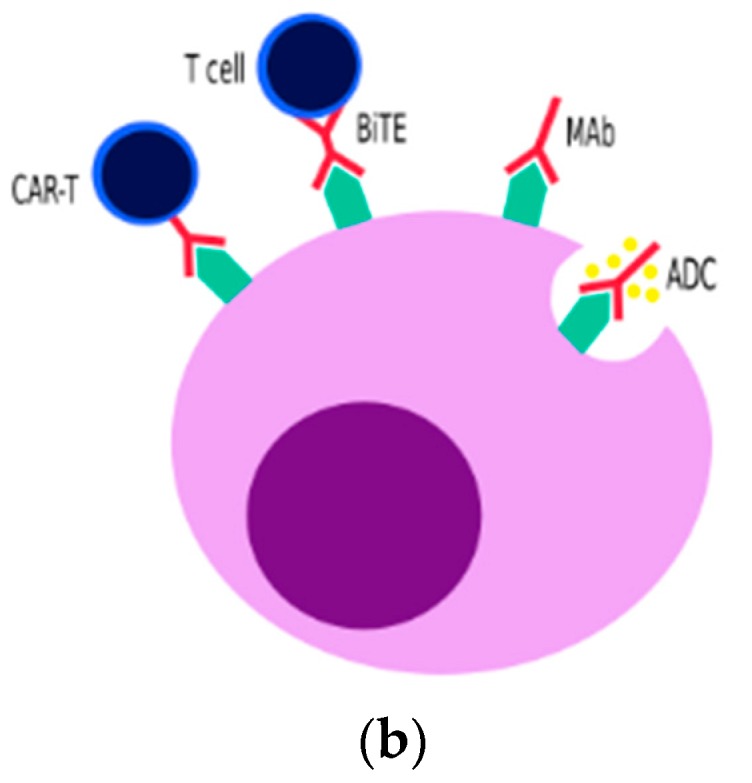
Overview of Immunotherapy in Multiple Myeloma. (**a**) Overview of different types of immunotherapy in MM. (**b**) Overview of how different types of immunotherapy may target the same antigen on a myeloma cell.

Dysfunctional host immune responses in MM raises a concern regarding the efficacy of vaccine-based MM therapeutics. The use of novel strategies to augment the host immune response against the tumor antigen may be of particular relevance to MM. MM cells engineered to express granulocyte-macrophage colony-stimulating factor (GM-CSF) such as GVAX recruits antigen presenting cells more effectively and represents an attractive modality for further study [211]. The combination of MM vaccines with monoclonal antibodies, as well as their use in the maintenance setting combined with checkpoint inhibitors, are also worthy of investigation in prospective clinical trials. The overview of immunotherapy in MM is summarized in Figure 1.

## 10. Clinical Application and Future Directions

It is now clear that immunotherapy is an integral part of MM management and ongoing efforts are taken to further refine this.

### 10.1. Monoclonal Antibodies as Frontline Treatment

Based on the promising results in phase 3 studies mentioned earlier, the use of daratumumab upfront is likely to be the standard of care, with combination of monoclonal antibody, proteasome inhibitor, IMiD and steroid being the future of induction therapy in MM.

The promise of improved PFS and increased MRD-negativity rates have put the myeloma treatment paradigm in great excitement. However, daratumumab has not really been proven to benefit the high-risk patients [28,68,69]. In terms of safety, the addition of daratumumab is also associated with increased risk of infection [28,68,69]. 

Studies have also shown feasibility of subcutaneous daratumumab [212], which will improve patients’ convenience as the intravenous daratumumab has quite long infusion duration. With the use of daratumumab as frontline treatment, the role of daratumumab as maintenance therapy is also being discussed. The CASSIOPEIA has a second randomization arm with and without maintenance daratumumab, which is still ongoing [68].

### 10.2. Ideal Sequencing of Immunotherapies

As the current trend is in using more daratumumab earlier in the disease trajectory, thoughts have to be put on to which drugs might be effective when these daratumumab-exposed patients relapse.

A small retrospective study showed that daratumumab re-treatment might be an option in daratumumab-refractory setting, although it might be confounded by the effect of daratumumab intensification used by the physicians in this retrospective analysis [213]. Although there is no concrete evidence of the benefit of daratumumab re-intensification in daratumumab monotherapy-refractory patients, it is commonly practiced in the clinical setting. On the other hand, reintroduction of a previously failed IMiD while keeping daratumumab as a backbone, without changing its dose or schedule has been shown to induce some efficacy which supported the concept of synergism between IMiDs with daratumumab [214].

ADC, BiTE, and CAR-T cell therapy are emerging treatment options in this era. The promising result of belantamab mafodotin and AMG420 clinical trials might suggest that these therapies are in the pipeline for future MM treatment paradigm. 

Early phase clinical trials using CAR-T cell therapy in the treatment of relapsed and/or refractory MM have also shown promising results, as mentioned earlier. There has been a great focus on targeting BCMA which is consistently expressed on myeloma cells and at low level in healthy, differentiated B cells, which is known to promote survival and proliferation of myeloma cells. Other targets, which includes CD19, kappa light chain, CD38, CD138, and SLAMF7, are also being explored, as discussed in the previous section.

Myeloma Genome Project analysis by Walker et al. showed that patients with double-hit had significantly poorer outcome as compared with patients with standard risk disease even when treated with novel agents, with survival of less than 2 years [215]. An analysis of patient outcome in the Myeloma IX trials also showed that patients with 2 or more high-risk genetics in combination with ISS stage 2 or 3 MM has median PFS of only 20 months, and strikingly lower as compared to patients without high-risk genetics and ISS stage I with median PFS of 50 months (*p* < 0.0001) [216]. As much as it has been shown that CAR-T cell therapy has great promise in the treatment of heavily pre-treated patients, it might possibly be useful if used earlier in high-risk group of patients in view of the poor outcomes with current treatment. This is also supported by the notion that heavily pretreated relapsed and/or refractory MM patients might have dysfunctional T cells, hence making CAR-T cell therapy less effective.

### 10.3. Durability of Response with CAR-T Cell Therapies

As much as we are excited with the excellent response seen with CAR-T cell therapy, it is not an effective cure as even patients with the best initial responses remain at high risk of disease progression. For example, among patients who achieved good responses to bb2121, a significant number of patients have progressed, including the ones who achieved MRD negativity [153]. These results show that CAR-T cells have potent cytoreductive capacity in MM but unlike in anti-CD19 CARs in acute lymphoblastic leukemia (ALL) or diffuse large B cell lymphoma (DLBCL), it may not provide long-term immune surveillance against relapsed MM and may not render MM curable. The postulated mechanism includes the emergence of BCMA-negative and positive relapse. The supportive evidence of short duration of remission as reflected in the median PFS of less than 1.5 year [151,152,153] despite excellent ORR of more than 80% in the CAR-T trials is particularly concerning. 

Efforts have been ongoing in continuous development of improved CAR-T therapies and strategies to mitigate this issue. One of the possible mechanism is the heterogenous expression of BCMA on myeloma cells. BCMA may not be universally expressed in every single myeloma cell. In this regards, targeting a combination of antigens expression on myeloma cells may be more effective. A single-arm study evaluating the combination of humanized anti-CD19 and anti-BCMA CAR-T cells in patients with relapsed or refractory MM in 21 patients in China showed that the approach of targeting different antigens separately is feasible [217]. The ORR was 95% with 9 (43%) sCRs and 3 (14%) CRs. Although most patients had CRS (90%), most (86%) were grade 1–2 [217].

Single CAR-T compound targeting 2 different antigens (bi-specific) have also been developed. A clinical trial evaluating a dual-target BM38 CAR incorporating both anti-CD38 and anti-BCMA single-chain variable fragment in tandem plus 4-1BB (CD137) signaling and CD3 zeta domains for relapsed and/or refractory MM is currently ongoing in China [218]. The preliminary result of 16 patients showed ORR of 87.5% with 50% sCR and 87.5% reached bone marrow MRD-negative status. The longest duration of sCR was over 51 weeks and 62.5% patients still maintained sCR (5 out of 8 patients), although 2 had transformed to VGPR and 1 to PR. PFS rates at 9 months was 75% [218].

The clinical study evaluating BCMA 2+1 T Cell Engager (TCE) CC-93269 has shown promising interim results with 83.3% ORR in heavily-pretreated relapsed and/or refractory MM. The concept of CC-93269 is in using an asymmetric 2-arm humanized IgG TCE that binds bivalently to BCMA and monovalently to CD3ε in a 2+1 format, so it would mediate interaction between T cells and BCMA-expressing myeloma cells to induce T cell receptor/CD3 crosslinking leading to T cell activation, and release of proinflammatory cytokines and cytolytic enzymes, resulting in myeloma cell death [219].

As BCMA is actively cleaved away from myeloma cell surface by the gamma-secretase complex, study has shown that small-molecule gamma-secretase inhibitor was able to increase BCMA expression on myeloma cells, hence able to increase CAR T-cell efficacy [220]. Combining CAR-T cell therapy with immunomodulatory drug [221] or checkpoint inhibitor [222] are interesting options to be explored.

A study evaluating dual targeting of BCMA and G protein–coupled receptor, class C group 5 member D (GPRC5D) with CAR-T cell therapy showed it could mitigate BCMA escape-mediated relapse in a xenograft model of MM. While parallel infusion of separate BCMA- and GPRC5D-targeted CAR T cells is effective, a single bi-cistronic vector encoding two 4-1BB-containing CARs avoids the practical challenges of parallel manufacturing, and uniquely provide superior anti-MM efficacy [223].

A novel dual-specific trimeric APRIL-based CAR targeting BCMA and Transmembrane activator and CAML interactor (TACI) is another potential for the future. TACI is almost solely expressed on plasma cells and found at high levels on most myeloma cells. By preserving the trimeric structure of APRIL, a proliferation-inducing ligand for both BCMA and TACI, it would allow enhanced binding to BCMA and TACI, which would in turn allow efficient targeting of both BCMA^+^ and BCMA^−^MM [224]. Further studies would be needed.

Efforts have also been made to increase the number of memory T cells with the aim to improve the persistence of CAR-T cells, which has been deemed as one of the mechanism of relapse post CAR-T cell therapy [225]. In line with the effort to enhance T cell activation and tumor targeting, an in vivo study of a tri-specific antibody targeting CD38, CD3 and CD28 has shown ability to suppress myeloma growth in a humanized mouse model and to stimulate memory/effector T cell proliferation and reduce regulatory T cells in non-human primates at well-tolerated doses [226].

Ultimately, to achieve longer effect and potential cure, the best time to use CAR-T could be during the earlier phases of the disease, for example, consolidation of high-risk patients with residual disease after initial standard treatment or at first relapse, rather than after many lines of treatment.

### 10.4. Logistic Challenges with CAR-T Cell Therapies

The source of T cells for manufacturing of CAR-T cell therapy has historically been the patient’s own T-cells which are harvested and engineered to target specific antigen/s before infusing it back to the patient. One of the challenges with this procedure is that the patient’s disease might progress during this process and might then not be fit to receive the CAR-T therapy, as it takes time to harvest cells and produce the CARs. There is also a possibility that the number of T cells harvested might not be enough, or are dysfunctional [227]. Recent development has tried to develop ‘off the shelf’ allogenic CAR-T cell therapies to mitigate these issues. The UCARTCS1 clinical trial, MELANI-01, which is the first allogeneic off-the-shelf CAR-T product candidate cleared by FDA, targets CS1 and is currently ongoing.

Allogenic CAR-T cell therapy is not without its own challenge, in particular, the development of graft-versus-host disease (GVHD) and the possibility of rejection. Hence, studies have been developed to evaluate the possibility of using allogenic NK-cells instead of T cells. The prospect of using allogenic CAR-NK cell therapies is based on the reasons that allogenic NK-cells should not cause GVHD and also NK-cells have limited life-span and therefore will be able to deliver effective antitumor activity while reducing the risk of long-term adverse effects. They also have intrinsic ability to target tumor cells via their native receptors [228]. On the same line, other ‘off-the-shelf’ solutions such as ADC and BiTEs, as described earlier, offer attractive options. Therefore, the pros and cons of the different immunotherapeutic approaches should be considered carefully based on factors such as cost, short- and long-term effectiveness, short- and long-term toxicities, state of patient’s immune system, and patient’s disease factors (e.g., high risk versus standard risk). The possibility of using these approaches in sequence or combinations should also be studied.

### 10.5. Toxicity with CAR-T Cell Therapies

With regards to toxicity, CRS is the main toxicity seen in CAR-T cell therapy in MM. Neurotoxicity in MM CAR-T cell therapy seems less severe as compared with that observed in B-ALL and DLBCL [229]. Most of the CRS were of grade 1 or 2. It can be difficult to compare rates of high-grade CRS between studies due to the use of different grading scales. The vast majority of severe (grades 3 and 4) CRS and neurotoxicity events were effectively treated with tocilizumab and supportive care, and patients recovered uneventfully.

### 10.6. Future Directions

With all the exciting development of immunotherapies, there are still many unanswered questions. As we have seen relapse which happens after CAR-T cell infusion, the debate is whether second CAR-T infusion is feasible and useful. It has been shown that BCMA expression increases during disease progression, suggesting that additional BCMA-targeted therapies might be beneficial [225].

Another thought is if maintenance is needed after CAR-T cell therapy to prevent relapse. It also remains unanswered if consolidation is needed after CAR-T cell therapy, for example, with allogenic stem cell transplantation. With the desirable CAR-T cell efficacy and unwanted long-term adverse effect, it has been questioned if it is possible to turn CAR-T on and off at different timing as programmed. 

With the ongoing success of immunotherapy, trial incorporating gene editing has emerged. A first-in-human trial using CRISPR-Cas9 technology to eliminate endogenous TCR and PD-1 and engineering of autologous T cells expressing NY-ESO-1 TCR has reported its preliminary result. Treatment of two heavily-pretreated relapsed refractory myeloma patients showed that it is feasible, with viable, expanding, and persisting CRISPR/Cas9 gene edited T cells that trafficked to tumor [230].

## 11. Conclusions

This era is an exciting and productive time in the field of immunotherapy for MM. With these many options, the outcome and survival of myeloma patients are expected to continue to improve. Based on the current advancement and trial results, the future of MM treatment paradigm will likely to include the use of monoclonal antibody upfront, in combination with potent novel agents, as well as the use ADC, BiTE, and CAR-T cell therapy in early relapse. Whether this will translate into eventual cure of myeloma remains an interesting debate and observation.

## Figures and Tables

**Table 1:** Monoclonal Antibodies.

Biologicals and Drugs	Disease Type	Recruited (Evaluable)	Efficacy Data	AE Grade 3–4 [SAE]	Trial Registration (Phase)	Reference
**Daratumumab (Anti-CD38)**						
Daratumumab, Bortezomib, Dexamethasone vs. Bortezomib, Dexamethasone (CASTOR)	RRMM	N = 498	ORR: 83.8% vs. 63.2% ≥VGPR: 62% vs. 29% mPFS 16.7 vs. 7.1 mo (DVd vs. Vd)	45.7% vs. 32.9% (DVd vs. Vd)	NCT02136134 (3)	[65,66]
Daratumumab, Lenalidomide, Dexamethasone vs. Lenalidomide, Dexamethasone (POLLUX)	RRMM	N = 569	ORR: 92.9% vs. 76.4% ≥CR: 51.2% vs. 21% mPFS NR vs. 17.4 mo (DRd vs. Rd)	88.7% vs. 76.9% (DRd vs. Rd)	NCT02076009 (3)	[29,83]
Daratumumab, Carfilzomib, Dexamethasone	RRMM	N = 85	ORR: 84%, ≥VGPR 71%	77% [45%]	NCT01998971 (1b)	[84]
Daratumumab, Pomalidomide, Dexamethasone	RRMM	N = 103	ORR: 60% mPFS 8.8 mo mOS 17.5 mo	78%	NCT01998971 (1b)	[85]
Atezolizumab (PD-L1), Daratumumab, Lenalidomide/Pomalidomide	RRMM	N = 24	≥VGPR: 53%	49% (0–100%)	NCT02431208 (1b)	[85]
Selinexor, Daratumumab, and Dexamethasome	RRMM	N = 30 (28)	ORR: 77%, ≥VGPR: 32.1%	66% [NA]	(1b)	[86]
Daratumumab, Bortezomib, Cyclophosphamide, Dexamethasone (LYRA)	NDMM, RRMM	N = 101	ORR 79% (ND), 71% (RR) VGPR: 44% (ND) 57% (RR) 12 mo PFS (87%), 12 mo OS (99%)	56% [21%]	NCT02951819 (2)	[87]
Daratumumab, Bortezomib, Melphalan, Prednisone vs. Bortezomib, Melphalan, Prednisone (ALCYONE)	NDMM	N = 737	ORR: 90.9% vs. 73.9%, ≥VGPR 72.9% vs. 49.7%, mPFS NR vs. 19.1 (DVMP vs. VMP)	39.9% vs. 38.7% (D-VMP vs. VMP) [NR]	NCT02195479 (3)	[67]
Daratumumab, Bortezomib, Thalidomide, Dexamethasone vs. Bortezomib, Thalidomide, Dexamethasone (CASSEOPEIA)	NDMM	N = 1085	CR: 39% vs. 26% (DVTd vs. VTd)	28% vs. 15% [NA]	NCT02541383 (3)	[68]
Ixazomib, Lenalidomide, Dexamethasone, Daratumumab	NDMM	N = 40 (38)	ORR: 95%, ≥VGPR: 58%	42%	NCT03012880 (2)	[88]
Daratumumab, Lenalidomide, Bortezomib, Dexamethasone vs. Lenalidomide, Bortezomib, Dexamethasone (GRIFFIN)	NDMM	N = 222 (16)	ORR: 100%, ≥VGPR: 100%	88%	NCT02874742 (2)	[89]
Ixazomib, Daratumumab, Dexamethasone	NDMM	N = 32 (10)	ORR: 70%, VGPR: 20%	NA, 82% (unfit) 88% (frail)	NTR6297	[90]
Daratumumab plus Lenalidomide and Dexamethasone for Untreated Myeloma (MAIA)	NDMM	N = 737	ORR: 92.9% vs. 81.3%, CR; 47.6% vs. 24.9% (DRd vs. Rd)	50% vs. 35.3% (62.9% vs. 62.7%) (DRd vs. Rd)	NCT02252172 (3)	[28]
**Isatuximab (anti-CD38)**						
Isatuximab	RRMM	N = 84	ORR: 23.8%	58% (43%)	NCT02514668	[91]
Isatuximab, Pomalidomide, Dexamethasone vs. Pomalidomide, Dexamethasone	RRMM	N = 307	ORR: 60.4% vs. 35.3%, ≥VGPR: 31.8% vs. 8.5%, mPFS 11.5 vs. 6.5 mo (IsaPd vs. Pd)	86.8% vs. 70.5% [NR}	NCT02990338 (3)	[92]
Isatuximab, Pomalidomide, Dexamethasone	RRMM	N = 34	NA	39.4% (8.8%)	NCT02283775 (1b)	[93]
Isatuximab, Bortezomib, Lenalidomide, Dexamethasone	NDMM	N = 22 (14)	ORR: 93%	46%, 18%	NCT02513186	[94]
**MOR202 (anti-CD38)**						
MOR202 monotherapy	RRMM	N = 44	ORR: 28%	64% (0%)	(1/2)	[95]
MOR202, dexamethasone vs. MOR202, Pomalidomide, Dexamethasone vs. MOR202, Lenalidomide, Dexamethasone	RRMM	N = 56	ORR: 28% (+dex), 65% (+len/dex), 48% (+pom/dex)	NA	NCT01421186 (1/2a)	[96]
**Anti-SLAMF7 antibody**						
Elotuzumab monotherapy	RRMM	N = 34	ORR: 0%	(44.1%)	(1)	[76]
Elotuzumab, Lenalidomide, Dexamethasone	RRMM	N = 73	ORR: 84%	78% [NA]	NCT00742560 (1b/2)	[97]
Elotuzumab, Lenalidomide, Dexamethasone vs. Lenalidomide, Dexamethasone	RRMM	N = 646	ORR: 79% vs. 66%, 3-year PFS 41% vs. 27%, mOS 43.7 vs. 39.6 mo (ERd vs. Rd)	78% vs. 67% (ERd vs. Rd)	NCT01239797 (3)	[77]
Elotuzumab, Pomalidomide, Dexamethasone vs. Pomalidomide, Dexamethasone	RRMM	N = 117	ORR: 53% vs. 26%, mPFS 10.3 vs. 4.7, mOS NR vs. 17.4 mo (EPd vs. Pd)	57% vs. 60% (EPd vs. Pd)	NCT02654132 (3)	[79]
Elotuzumab, Thalidomide, Dexamethasone vs. Thalidomide, Dexamethasone	RRMM	N = 40	ORR: 38%, mPFS 3.9 mo, mOS 16.3 mo	63% [NR]	NCT01632150 (2)	[98]
Elotuzumab, Bortezomib, Dexamethasone vs. Bortezomib, Dexamethasone	RRMM	N = 152 (150)	ORR: 66% vs. 63% VGPR: 36% vs. 27%, mPFS 9.7 vs. 6.9 mo (EVd vs. Vd)	71% vs. 60% [51% vs. 41] (EVd vs. Vd)	NCT01478048 (2)	[99]
**Anti-IL6 antibody**						
Siltuximab (CNTO328), Bortezomib vs. Bortezomib	RRMM	N = 281	ORR: 55% vs. 47% mOS 30.8 vs. 36.8 mo (SB vs. B), no efficacy	49% vs. 29% (SB vs. B)	NCT00401843 (2)	[80]
Siltuximab, dexamethasone	RRMM	N = 55 (53)	ORR 19%	36% [0]	Phase 2	[81]
Siltuximab, Bortezomib, Melphalan, Prednisolone vs. Bortezomib, Melphalan, Prednisolone	NDMM	N = 106	CR: 27% vs. 22% (SVMP vs. VMP), did not meet primary endpoint	92% vs. 81% (SVMP vs. VMP)	NCT00911859 (2)	[100]
Siltuximab, Bortezomib, Lenalidomide, Dexamethasone	NDMM	N = 11	ORR: 90.9%, ≥VGPR: 45.5%	63.6% [9.1%]	NCT01531998 (1/2)	[101]
**Anti-CD138 antibody**						
Indatuximab ravtansine (BT062)	RRMM	N = 29 (23)	≥SD: 50%	Grade 1/2: 90%	(1/2)	[102]
Indatuximab ravtansine (BT062), lenalidomide	RRMM	N = 45	ORR: 83%	Grade 1/2: 89%	NCT01638936 (1/2a)	[103]
Indatuzimab ravtansine, Lenalidomide or Pomalidomide, Dexamethasone	RRMM	N = 64 (43)	ORR: 77%	Grade 1/2: 90%	NCT01638936 (1/2a)	[104]
**Anti-CD56 antibody**						
Lorvotuzumab Mertansine (IMGN901) monotherapy	RRMM	N = 37 (35)	≥SD: 42.9%, PR: 5.7%	5.4% [0]	NCT00346255 (1)	[105]
Lorvotuzumab mertansine, Lenalidomide, Dexamethasone	RRMM	N = 44 (41)	ORR: 59%	NA	NCT00991562 (1/2)	[106]
**Others**						
ACTR087, SEA-BCMA	RRMM	N = 2	50% (1/2)	NCT03266692 (1)	[107]
Tabalumab, Bortezomib, Dexamethasone	RRMM	N = 220	ORR: 58.1% vs. 59.5% vs. 61.1%, mPFS 6.6,7.5 vs. 7.6 mo (tabalumab 100, tabalumab 300 vs. placebo)	37% [9.1%]	NCT01602224 (2)	[82]

**Table 2:** CAR-T cell therapies, ADC, and BiTE.

Study Title	Target	Recruited (Evaluable)	Efficacy Data	CRS Grade 3–4 [Neurotoxicity]	Trial Registration	Reference
bb2121 anti-BCMA CAR T-Cell Therapy in Patients with Relapsed/refractory Multiple Myeloma: Updated Results from a Multicenter Phase I Study	BCMA	N = 33	ORR: 85%, CR: 45%, mPFS: 11.8 mo	5% [2%]	NCT02658929	[156]
Safety and Efficacy of B-cell Maturation Antigen (BCMA)-Specific Chimeric Antigen Receptor T Cells (CART-BCMA) with Cyclophosphamide Conditioning for Refractory Multiple Myeloma (MM)	BCMA	N = 25	ORR: 64% for the highest dose	32% [12%]	NCT02546167	[157]
Updated Analysis of a Phase 1, Open-Label Study of LCAR-B38M, a Chimeric Antigen Receptor T Cell Therapy Directed Against B-Cell Maturation Antigen, in Patients with Relapsed/Refractory Multiple Myeloma	BCMA	N = 57	ORR: 88%, CR: 68%, mPFS: 15 mo at median 8mo f/u	7% [0%]	NCT03090659	[158]
Initial Results from a Phase 1 Clinical Study of bb21217, a Next-Generation Anti BCMA CAR T Therapy	BCMA	N = 7	ORR: 86%, CR: 25%	14% [14%]	NCT03274219	[159]
Fully Human BCMA Targeted Chimeric Antigen Receptor T Cells Administered in a Defined Composition Demonstrate Potency at Low Doses in Advanced Stage High Risk Multiple Myeloma	BCMA	N = 7	ORR: 100%	0% [0%]	NCT03338972	[160]
Efficacy and Safety of P-Bcma-101 CAR-T Cells in Patients with Relapsed/Refractory (r/r) Multiple Myeloma (MM)	BCMA	N = 23	ORR: 81%	0% [4.8%]	NCT03288493	[161]
Clinical Reponses and Pharmacokinetics of Fully Human BCMA Targeting CAR T Cell Therapy in Relapsed/Refractory Multiple Myeloma	BCMA	N = 9	ORR: 100%, CR: 67%	0.2% [0%]	ChiCTR1800018137	[162]
Improved Efficacy and Safety of a Dual-Target CAR-T Cell Therapy Targeting BCMA and CD38 for Relapsed/refractory Multiple Myeloma	BCMA and CD38	N = 12	ORR: 83.3%, ≥VGPR: 58.3%	33.3% [0%]	ChiCTR1800018143	[163]
Clinical Responses and Pharmacokinetics of MCARH171, a Human-Derived Bcma Targeted CAR T Cell Therapy in Relapsed/Refractory Multiple Myeloma: Final Results of a Phase I Clinical Trial	BCMA	N = 11	ORR: 100%	20% [0%]	NCT03070327	[164]
Durable Remission Achieved from BCMA-Directed CAR-T Therapy Against Relapsed or Refractory Multiple Myeloma	BCMA	N = 17	ORR: 79%	7% [7%]	NCT03093168	[165]
JCARH125, Anti-BCMA CAR T-cell Therapy for Relapsed/Refractory Multiple Myeloma: Initial Proof of Concept Results from a Phase 1/2 Multicenter Study (EVOLVE)	BCMA	N = 19	ORR: 100%, CR: 67%	0% [12.5%]	NCT03430011	[166]
T Cells Genetically Modified to Express an Anti-B cell Maturation Antigen Chimeric Antigen Receptor Cause Remissions of Poor-Prognosis Relapsed Multiple Myeloma	BCMA	N = 24	ORR: 81%	38% [19%]	NCT02215967	[151]
Combined Infusion of CD19 and Bcma-Specific Chimeric Antigen Receptor T cells for RRMM: Initial Safety and Efficacy Report from a Clinical Pilot Study	BCMA/CD19	N = 8	ORR: 80%	12.5% [0%]	NCT03196414	[167]
Tandem Autologous Transplantation and Combined Infusion of CD19 and Bcma-Specific Chimeric Antigen Receptor T Cells for High Risk MM: Initial Safety and Efficacy Report from a Clinical Pilot Study	CD19/BCMA	N = 9	ORR: 100%, CR: 51%	0% [0%]	NCT03455972	[168]
Low Dose of Human scFv-Derived BCMA-Targeted CAR-T Cells Achieved Fast Response and High Complete Remission in Patients with Relapsed/Refractory Multiple Myeloma. Jiang S et al. Blood 2018 132 960	BCMA	N = 16	ORR: 100%	6% [0%]	NA	[169]
T Cells Expressing Anti B-cell Maturation Antigen Receptors for Plasma Cell Malignancies	BCMA	N = 28	ORR: 87%	14% [0%]	NA	[170]
B-Cell Maturation Antigen Antibody-Drug Conjugate (ADC), GSK2857916, in Relapsed/refractory Multiple Myeloma (RRMM): Final Safety, Efficacy and Pharmacokinetic (PK) Analyses from a Phase I Study	BCMA	N = 73	ORR: 60%, mPFS 12 mo	20% [NA]	NA	[171]
Pilot Study of Anti-CD19 Chimeric Antigen Receptor T Cells (CTL019) in Conjunction with Salvage Autologous Stem Cell Transplantation for Advanced Multiple Myeloma	CD19	N = 10	ORR: 20%	0% [0%]	NCT2135406	[172]
CD138-directed Adoptive Immunotherapy of Chimeric Antigen Receptor (CAR)-Modified T Cells for Multiple Myeloma	CD138	N = 5	-ORR: 80%	0% [0%]	NCT01886976	[173]
Clinical Responses with T lymphocytes Targeting Malignancy-associated κ Light Chains	Kappa LC	N = 7	ORR: 0%	0% [0%]	NCT00881920	[145]
Safety and Efficacy of Multiantigen-Targeted T Cells for Multiple Myeloma	PRAME, SSX2, MAGEA4, NY-ESO-1 and Survivin	N = 18 (10)	CR: 10%	NA	NA	[174]
Evaluation of AMG 420, An Anti-BCMA Bispecific T-Cell Engager (BITE®) Immunotherapy, In R/R Multiple Myeloma (MM) Patients. Updated Results of a First-In-Human (FIH) Phase 1 Dose Escalation Study	BCMA BITE	N = 42	ORR: 70%, ≥VGPR: 14.2%	Grade 2–3 CRS 7.1%	NCT02514239	[163]
